# 2244 The effects of gravidity and parity on risk of cognitive impairment and amyloid plaque deposition

**DOI:** 10.1017/cts.2018.188

**Published:** 2018-11-21

**Authors:** Rebecca DiBiase, Aozhou Wu, David Knopman, Keenan Walker, Thomas Mosley, Pamela L. Lutsey, Rebecca Gottesman

**Affiliations:** 1 Johns Hopkins University School of Medicine; 2 Johns Hopkins Bloomberg School of Public Health; 3 Mayo Clinic; 4 University of Mississippi Medical Center; 5 University of Minnesota School of Public Health; 6 Johns Hopkins University School of Medicine and Bloomberg School of Public Health

## Abstract

OBJECTIVES/SPECIFIC AIMS: Our study seeks to answer the following questions: (1) To determine whether higher numbers of gravidity and parity are associated with a decreased risk of mild cognitive impairment or dementia; (2) To determine whether higher numbers of gravidity and parity are associated with a decreased risk of amyloid deposition by PET MRI. METHODS/STUDY POPULATION: Our study population includes all female study participants in the Atherosclerosis Risk in Communities (ARIC) study who did not have a diagnosis of dementia before enrollment. Participants were also required to have been evaluated for cognitive impairment in the ARIC-NCS ancillary study, or to have received an MRI PET scan of their brain as part of the ARIC-PET ancillary study. Baseline information on the gravidity and parity of all the women was recorded at initial enrollment. We use statistical analyses and epidemiological measures to explore our study questions. For our first question, we use logistic regression to evaluate the association of gravidity and parity as two separate ordinal variables using adjudicated mild cognitive impairment (MCI) and dementia. For our second question, we use logistic regression to evaluate the association of gravidity and parity (again as ordinal variables) with amyloid positivity. We use STATA for our statistical analyses. RESULTS/ANTICIPATED RESULTS: We hypothesize that increased gravidity and parity will have either no effect or a protective effect against MCI, dementia, and amyloid deposition. Our preliminary analyses show that older age of a woman at first pregnancy and at first live birth are both positively correlated with increased incidence of cognitive impairment. No relationship was found between these surrogates of lifetime estrogen exposure and cerebral amyloid deposition. DISCUSSION/SIGNIFICANCE OF IMPACT: Multiple basic science and clinical research studies have shown that estrogen exposure has an effect on cognitive function, likely through a complex interplay of multiple physiologic systems. Our study expands the research in this area by using a large, established epidemiologic cohort to examine gravidity and parity as important factors in lifetime estrogen exposure as they relate to cognitive impairment and amyloid plaque deposition.

